# Perceptions, beliefs, and needs of Japanese people with knee osteoarthritis during conservative care: a qualitative study

**DOI:** 10.1186/s12891-021-04641-7

**Published:** 2021-09-03

**Authors:** Daisuke Uritani, Akane Ikeda, Toru Shironoki, Kentaro Matsubata, Yuto Mutsura, Tadashi Fujii, Koji Ikeda

**Affiliations:** 1 Department of Physical Therapy, Faculty of Health Science, Kio University, 4-2-2, Umaminaka, Koryocho, Kitakatsuragigun, Nara, 6350832 Japan; 2 Department of Orthopaedic surgery, Kashiba Asahigaoka Hospital, 839, Kaminaka, Kashiba city, Nara, 6390265 Japan; 3 Department of Rehabilitation, Faculty of Health Science, Naragakuen University, 3-15-1, Nakatomigaoka, Nara city, Nara 6318524 Japan

**Keywords:** Osteoarthritis, Knee, Qualitative study, Patient care management

## Abstract

**Background:**

Patients’ perceptions and beliefs of disease could be influenced by their lifestyle and culture. Although it is important to understand their perceptions and beliefs toward disease to prevent and manage osteoarthritis (OA) through conservative care, this topic has not been investigated in Japanese people with knee OA. Therefore, this qualitative study aims to clarify how Japanese patients with knee OA experience and perceive their symptoms and disabilities, and how they face them during conservative care.

**Methods:**

Participants were recruited by purposive sampling. Face-to-face, semi-structured interviews were conducted with nine patients (2 men and 7 women; mean age, 74.3 ± 5.5 years) with knee OA until data saturation was reached. Interview data comprised participants’ accounts of particular personal experiences of living with knee OA, including their perceptions and attitudes toward knee OA-related symptoms and disabilities. Two physiotherapists (one with extensive experience conducting qualitative studies) and four physiotherapy students conducted the interviews. Recorded interview data were transcribed verbatim in Japanese. Data analysis, including developing a coding scheme, was conducted based on a grounded theory approach.

**Results:**

Two core categories were extracted from the data: ‘Negative experiences’ and ‘Coping with difficulties’. ‘Negative experiences’ included three main categories: ‘Self-analysis on the cause of knee OA’, ‘Difficulties in daily life due to knee symptoms’, and ‘Psychological barrier’. ‘Coping with difficulties’ included three main categories: ‘How to deal with knee pain and difficulty in moving’, ‘Information considered useful to cope with knee OA’ and ‘Importance of connecting with others’. Japanese patients with knee OA desired evidence-based information and to connect with other people in the same situation to solve problems related to their condition.

**Conclusions:**

To address patients’ concerns, medical professionals should conduct careful interviews and obtain information regarding patients’ past experiences, and understand their experiences related to knee OA. Symptoms and difficulties experienced by patients with knee OA should be managed by evidence-based information integrating their perceptions and beliefs toward knee OA.

**Supplementary Information:**

The online version contains supplementary material available at 10.1186/s12891-021-04641-7.

## Background

Knee osteoarthritis (OA) is a prevalent, costly chronic condition characterised by physical symptoms and functional limitation in older people [1–3]. In Japan, more than 60% of adults aged 60 years or above suffer from radiographic knee OA, and more than 26% of adults aged 60 years or above have symptomatic knee OA [4]. Self-management, OA education, weight management, and regular exercise during conservative care are important for patients with knee OA to improve their condition [5, 6].

Using the tools available to them, healthcare professionals must understand how patients perceive knee OA and the feasibility of self-management of symptoms, and understand patients’ information-related needs about knee OA [7]. Understanding how people talk about knee OA and the potential implications of how they talk can inform health communication and health care [8]. At the same time, a therapist’s inability to understand how patients perceive their situation is a key barrier to successful public health activities [9, 10].

Patients’ perceptions and attitudes toward the disease reflect their experiences. How people talk about their health both shapes and reflects their experiences with and attributes of health, illness, and health care [11]. For example, people aged 35–65 years suffering from painful knees believed that OA’s progression could be prevented or delayed and did not consider OA a natural or inevitable event [12]. Meanwhile, people aged 60 years and above perceive OA as a naturally occurring part of ageing [13–15]. These findings suggest that patients with different backgrounds perceive knee OA differently.

Previous studies indicated that people’s attitudes toward dealing with pain [16], health beliefs, and self-care [17] varied due to differences in lifestyle and culture between Japan and other countries. Therefore, perceptions toward knee OA may also differ between Japan and other countries. For successful conservative management of patients with knee OA, healthcare professionals must understand patients’ perceptions and beliefs toward knee OA and their needs. Patients’ perceptions and attitudes toward knee OA have been reported and synthesised in Western and other Asian countries [18–20]. For example, previous studies indicated the importance of peer assistance and culturally specific activities [18], distrust in Western medicine [18], informal information gathering [19], and the physical, emotional, and social impact [20] to understanding these perceptions and attitudes. However, patients’ perceptions and attitudes toward knee OA have not been sufficiently explored in Japan.

In the conservative care of knee OA patients, not only medical information but also backgrounds, thoughts, and preferences must be managed from the patient’s perspective. Understanding the patients’ perceptions, beliefs, and needs better may lead to managing patients more effectively and result in better patient outcomes. Therefore, this qualitative study aimed to explore Japanese patients’ perceptions and beliefs towards knee OA and their needs during conservative care.

## Methods

This study employed a cross-sectional, qualitative design. It adhered to the standards for reporting qualitative research [21]. This study adopted a qualitative research approach because it not only aimed to summarise the data obtained from the interviews but also to develop a theory depicting the processes through which the phenomena occur and to elucidate the relationship between the phenomena illustrated by the data [22].

### Participants

Study participants comprised patients with knee OA from different regions in Japan. Inclusion criteria were a confirmed diagnosis of knee OA in one or both knees, aged over 50 years, and self-reported experience of pain or disability. Patients suspected of having cognitive decline or communication difficulties, although not specifically tested, were excluded. Purposive sampling according to the inclusion criteria determined by the research purpose was performed to obtain rich, relevant, and diverse data pertinent to the research question [23]. Japanese people are generally said to traditionally emphasise stoicism and conceal pain and emotions. A previous study reported that Japanese people considered pain behaviours, such as crying or showing pain, to be less acceptable than do Western people [16]. According to a large-scale survey, about two-thirds of Japanese people with chronic pain believed that they should tolerate their pain, and more than half of Japanese people with chronic pain believed that they should not feel free to tell others about their pain [24]. Especially in the first few interviews, we purposively sampled expressive people because we needed to generate a framework category from a wealth of narratives. Therefore, we purposively recruited patients who seemed extroverted to gather detailed and descriptive information. Furthermore, we recruited patients who had different backgrounds; for example, we included people who had received physical therapy and undergone total knee arthroplasty (TKA) and those who had not. Study participants were recruited by an orthopaedic surgeon or physical therapists at facilities that cooperated with this research and an author of this study (DU). Outpatients of cooperating facilities and people attending the health promotion program of DU’s university were asked to come to the clinic or the university to join the interview. We included patients who accepted to participate in this study. Permission to record the interviews and written informed consent were obtained from all participants before including them in the study.

### Interviews and data collection

The interviewers were two physiotherapists and four physiotherapy students (one woman and five men). All interviewers were Japanese. The two physiotherapists had 19 (DU) and 26 (KI) years of work experience in physiotherapy, including musculoskeletal physiotherapy. Physiotherapist KI was also an experienced qualitative researcher. The physiotherapy students (AI, TS, KM, YM) were in the final year of their baccalaureate degree program and had completed clinical clerkship in their undergraduate curriculum. Interviews were held in an isolated room at the orthopaedic clinic where we recruited participants and at a meeting room at DU’s university. Data were collected from February to August 2019. One interviewer (DU) conducted all interviews to ensure consistency with other researchers. The four physiotherapy students were trained by researcher KI in advance of the study. Participants were unknown to the interviewers prior to recruitment. Each interview lasted approximately 1–1.5 h. Participants were interviewed one at a time, except for two participants who were a couple, and face-to-face. We told participants to treat us as lay interviewers rather than experts. Interview data comprised participants’ experiences of living with knee OA to clarify the perceptions and beliefs toward symptoms and disabilities about knee OA and their needs to deal with their symptoms and difficulties during conservative care. Semi-structured interviews were conducted using an interview guide created based on previous studies [12, 25–28] (Table 1) and comprising open-ended questions to elicit participants’ perspectives of their experiences and ideas regarding knee OA. Guided questions encouraged participants to describe their experiences and thoughts regarding knee OA during conservative care. The interviews were kept flexible to allow participants to talk about what they deemed important. Additionally, interesting or important statements from participants interviewed previously were used as questions to subsequent participants. All the interviews were recorded using an IC recorder (ICD-SX950, SONY, Tokyo, Japan) and recorded data were managed using a software program (Sound Organizer Ver.1.6). Observational memos, including contextual characteristics, atmosphere, and relevant non-verbal expressions, were also recorded. No repeat interviews were conducted.

**Table 1:** Interview guide

*Introduction*	
Please tell us if you remember the first time you noticed the symptom (or pain).	
*Perceptions*	
How did you feel when you were diagnosed with knee osteoarthritis?	
What are your thoughts on your current symptoms (or pain)?	
Do you think this symptom (or pain) will remain in the future? Why do you think so?	
*Physical*	
What was the most important (or first) physical concern when you were diagnosed with knee osteoarthritis?	
How is your knee condition now?	
Do you want to reduce pain further with surgery?	
Have you ever been happy with the pain?	
Is there any difference between pain in other areas and knee pain?	
*Life*	
What was the most important (or first) concern in your life when you were diagnosed with knee osteoarthritis?	
How has knee osteoarthritis affected your daily life?	
Are you doing anything to mitigate those effects?	
*Information*	
What do or did you know about knee osteoarthritis?	
Where and how did you collect information on how to deal with illness and pain?	
What kind of information has been useful so far?	
What kind of information did you want at the time of your first visit (or onset)?	
What kind of information do you want now?	
*Others*	
What kind of services do you currently receive (hospitals, outpatients)?	
What kind of service are you looking for?	
With whom do you talk about your current symptoms (pain, etc.)?	
How do you explain your symptoms to others?	
Do you keep a record of your illness? Why?	
*Summary*	
If you could go back in time and do something differently to prevent or manage knee OA, what would it be?	
Do you have any other experience or feelings on this subject that you would like to talk about?	

Recruitment, data collection, and data analysis occurred concurrently to enable data to inform subsequent interviews and to cease recruitment once theoretical saturation was achieved. The criterion for saturation was that no new theme was identified in the new interview after reaching a consensus among the researchers. Recorded data were transcribed verbatim in Japanese after the interview by five interviewers (DU, AI, TS, KM, YM). Recorded data from each interview were divided into several parts. Vocal inflexions and utterances were indicated by the interviewer where necessary. The transcripts were crosschecked against the recorded data to ensure their completeness and accuracy by another interviewer.

### Analysis

Data analysis was conducted based on a grounded theory approach [29, 30]. The grounded theory approach was used to develop a theory about the process of phenomena-generation for the participants based on data generated from their narrative and the relationship between the phenomena from their narrative. Transcripts were divided into several sentences according to the content and context of the statements and coded individually. These coded parts were listed and clustered into groups to form initial small categories (subcategories). This was done to create draft categories for the data obtained from the interview of the first participant. After the interview of the second participant, the divided parts of the transcripts were coded, and already-formed draft categories were applied. These procedures formed an iterative process that involved integrating closely related codes, separating other more complex ones into separate elements, and developing new categories where necessary. Relationships between and within the small categories that emerged from this process were explored with increasingly higher levels of conceptualisation. Small categories across all transcripts were then listed and clustered into larger categories based on similarity and overlap. This grouping was refined to identify main themes. Coding and categorising were crosschecked by all researchers, with all disagreements resolved through regular discussions.

## Results

Interview data were obtained from nine participants with knee OA (mean age: 74.3 ± 5.5 years; 77.8% women; Table 2). Six participants were recruited from among outpatients at a general hospital, of which two participants were from an orthopaedic clinic, and one participant was from among attendees of a community-based health care program. Three participants had undergone TKA in the past. Participants who had undergone TKA were also interviewed about their experiences of conservative treatment before TKA.

**Table 2:** Demographic data for the nine participants involved in the study

ID	Sex	Age (years)	Symptom duration	Place recruited	Employment status	KL grade (R/L)	Pain^a^ (R/L)
A	W	70	3 mos	Hospital in suburban area	Housewife (real estate business retired)	4/0	5/0
B	M	67	5 yrs	Hospital in suburban area	Carpenter	TKA/TKA	1/1
C	M	77	10 yrs	Hospital in suburban area	Volunteer work (office worker retired)	3/3	4/4
D	W	75	10 yrs	Hospital in suburban area	Volunteer work, housewife	3/3	3/4
E	W	76	Over 25 yrs	Hospital in suburban area	Housewife (family operated business, retired)	TKA/4	0/5
F	W	83	3 mos	Clinic in urban area	Housewife	2/2	5/5
G	W	74	3 mos	Clinic in urban area	Housewife	1/1	2/2
H	W	67	25 yrs	Hospital in suburban area	Part-time university lecturer	TKA/TKA	1/1
I	W	80	1 yr	Health care program in suburban area	Housewife (medical processor, Tai Chi instructor, retired)	0/2	0/3

*KL grade* Kellgren-Lawrence grade, *KOOS* Knee injury and Osteoarthritis Outcome Score, *L* Left, *M* Man, *NRS* Numerical Rating Scale, *R* Right, *TKA* Total Knee Arthroplasty, *W* Woman

^a^Pain is described by the Numerical Rating Scale

Two core categories -‘Negative experiences’ and ‘Coping with difficulties’-were developed from the data. ‘Negative experiences’ included three main categories: ‘Self-analysis on the cause of knee OA’, ‘Difficulties in daily life due to knee symptoms’, and ‘Psychological barrier’. ‘Coping with difficulties’ included three main categories: ‘How to deal with knee pain and difficulty in moving’, ‘Information considered useful to cope with knee OA’, and ‘Importance of connecting with others’. In ‘Negative experiences’, ‘Psychological barrier’ resulted from negative emotions or cautious behaviour as a result of ‘Self-analysis on the cause of knee OA’ and ‘Difficulties in daily life due to knee symptoms’. As a way of ‘Coping with difficulties’, participants found ‘How to deal with knee pain and difficulty in moving’ independently, and they had ‘Information considered useful to cope with knee OA’ and recognised the ‘Importance of connecting with others’.

**Fig. 1:**
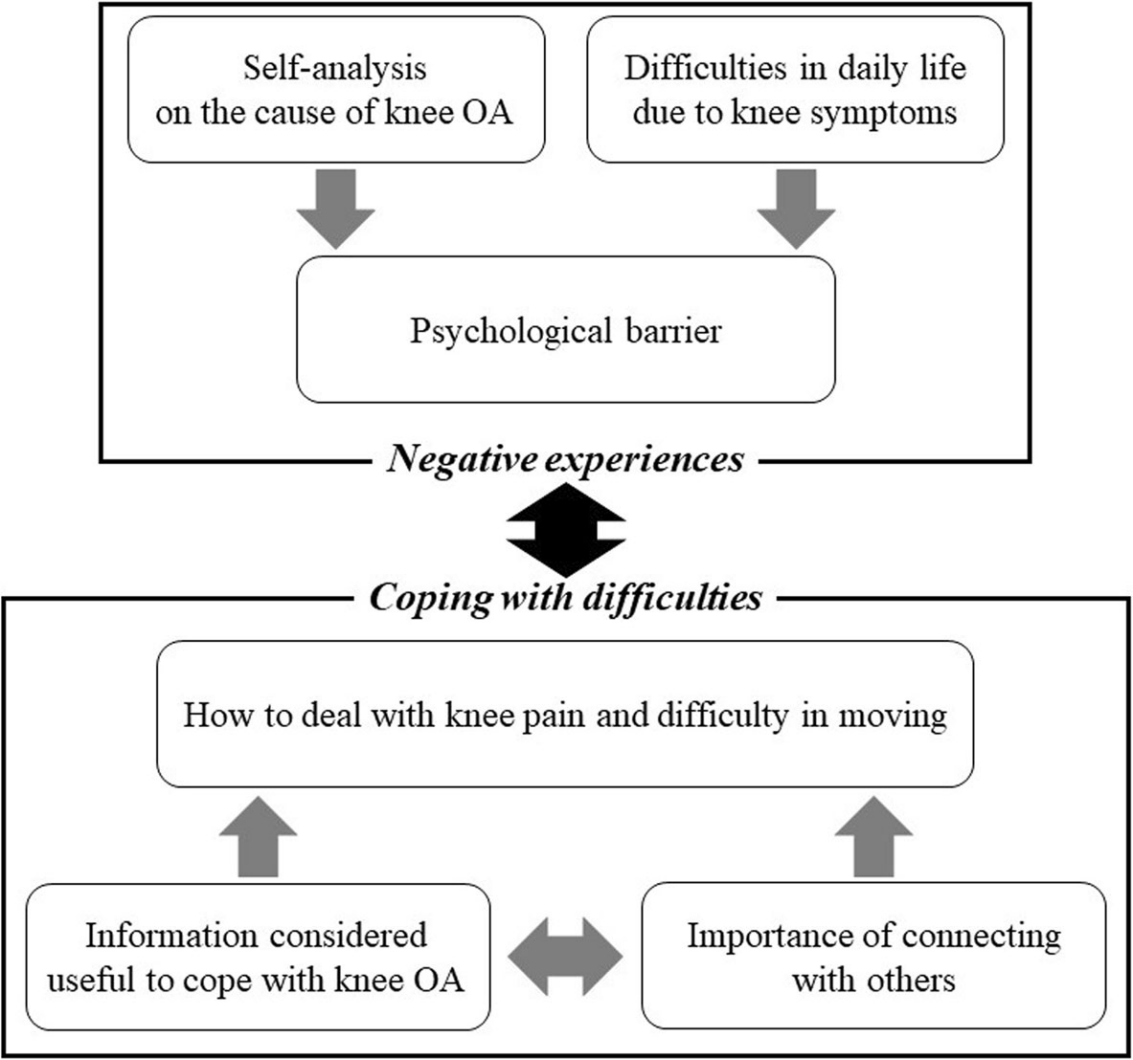
Relationship between categories. Core categories: ‘Negative experiences’ and ‘Coping with difficulties’. Under each core category, three main categories were created. ‘Negative experiences’ includes ‘Self-analysis on the cause of knee OA’, ‘Difficulties in daily life due to knee symptoms’ and ‘Psychological barrier’. ‘Coping with difficulties’ includes ‘How to deal with knee pain and difficulty in moving’, ‘Information considered useful to cope with knee OA’, and ‘Importance of connecting with others’

The main categories are presented below, followed by their subcategories. They are described with illustrative quotes presented throughout the text using pseudonyms that match Table 2. Supplemental illustrative quotes are given in the Additional file 1. Figure 1 depicts the relationship between categories.

### Core category: negative experiences

#### Self-analysis on the cause of knee OA

##### Overwork/overload

Participants believed that overworking their knees in their youth was responsible for their current knee condition. They suspected that the effects of other body parts, especially the condition of feet and ankles, or personal characteristics of walking and movement might be associated with their overloaded/overworked knee joint.

*‘I think I've overworked my knees. When I thought about various causes of knee OA ...I think I was walking more than others. I was guiding customers at work. That's the reason for knee OA that comes to mind’. (A)*

*‘I think that hallux valgus was progressing. So, I also think that the hallux valgus might have affected the knee’. (G)*

##### Ageing

All participants mentioned whether ageing was associated with the development or progression of knee OA. Some participants believed that ageing had caused knee OA, but others did not.

*‘I think there is an age effect (on knee OA). When I was diagnosed with knee osteoarthritis, I thought I was finally old’. (G)*

*‘I think many people believe that the cause of knee OA is ageing, but I don't think age is related to knee OA’. (E)*

##### Exposure to cold

A few participants suspected that exposure to cold might have contributed to deteriorating the condition of their knees. They cited their constitution, overcooling their knees due to air conditioning, and their inability to cope with temperature changes.

*‘I have a constitution that causes pain in my joints when my body gets cold’.* (E)

#### Difficulties in daily life due to knee symptoms

##### Movement with bending the knee

Participants described having difficulty in movements that required bending the knee. In the Japanese lifestyle, deeply bending the knee, such as for squatting or sitting with legs folded, is required on various occasions, and there were some cases where their movement was hindered.

*‘I can no longer sit down on my heels on the tatami mat’. (A)*

*‘It's very difficult to pick up objects on the floor with bent knees’. (I)*

##### Start of movement

Difficulty in initiating movement due to knee pain or stiffness are common symptoms in patients with knee OA. The participants in this study had similar complaints.

*‘When I wake up in the morning and get out of bed, I feel weak in my legs and I almost fall, or I feel pain in my knees’. (F)*

*‘When I stood for a long time and started moving, I found it difficult to move’. (H)*

#### Psychological barrier

##### Prudence about movement and activities

Participants were gradually becoming more cautious about their movements to prevent increasing pain and falls. More importantly, it made them cautious about going out and interacting with others.

*‘I always care about my knees. I walk while looking down and I am careful not to fall’. (E)*

*‘I think that the experience of being unable to move my legs or having a pain in my knees is a barrier not only to my behaviour but also to my mentality. That has led to restrictions on activities’. (H)*

##### Not wanting to bother others

Participants reported feeling sorry for the inconvenience caused to others by leaving their jobs or roles in the community. Additionally, they thought that pain and distress should not be complained about to others and should be tolerated.

*‘I had to take a break from working part-time and teaching Tai Chi. It was the hardest thing to bother people around me’. (I)*

*‘When I complain about my pain or trouble to others, I regret saying something people don't like to hear’. (G)*

##### Desire to avoid surgery

Participants expressed their desire to avoid surgery. Those who continue treatment conservatively have anxiety or fear and negative thoughts about surgery. The idea of patience as a virtue was also reflected in the idea of surgery.

*‘I'm wondering if I should undergo arthroplasty, but I'm trying to delay it as much as possible. If possible, I would like to avoid arthroplasty..’. (A)*

*‘It may be due to the characteristics of Japanese beliefs, but it cannot be denied that I regard undergoing surgery as a defeat’. (F)*

### Core category: coping with difficulties

#### How to deal with knee pain and difficulty in moving

##### Controlling activities based on subjective sensations

Participants relied on their physical sensations to control their amount of exercise and physical activity. Some participants made intuitive judgements. Others used pain as an indicator of the amount of exercise and activity.

*‘It depended on the day, but I could somehow judge (physically) that it was not good if I moved anymore’. (B)*

*‘When I go walking, I stop walking when I feel like I can't stand the pain’. (D)*

##### Continuing exercise and physical activity

Participants recognised the importance of exercise and physical activity. They participated in volunteer activities and actively engaged in personal activities, such as garden maintenance. Others walked or did strength training. While recognising the importance of these activities, the difficulty of performing them was also acknowledged.

*‘I will try to walk as much as possible so that my symptoms do not get worse. I am thinking of doing activities as long as I can move’. (I)*

*‘I affirm in my head that I have to exercise, but, in fact, it is difficult to put it into practice while reflecting on it’. (F)*

##### Ingenuity to reduce knee pain and difficulty in movement

Participants described their ingenuity in daily movements, especially in walking, to reduce knee symptoms. They also had other ideas regarding equipment use, such as handrails and chairs.

*‘I think that how to move and walk is important to alleviate symptoms of the knee. So, I'm trying to deal with knee symptoms by thinking about countermeasures myself’. (G)*

*‘I attached handrails to the bath and toilet and used chairs in the bathroom’. (B)*

*‘Since my knees can't bend, I sit in a chair with small casters when I work in the garden.’ (E)*

##### Way of thinking

Participants considered independence and a positive attitude toward life and activities important to face knee OA. They expressed that negative thoughts put them in a negative state and positive thoughts in a positive state.

*‘Through various experiences related to knee OA, I came to think that I had to have a positive way of thinking. I’m thinking, “I have to do my best. Let's do our best!”’ (E)*

*‘I think that personality is also very important when dealing with knee OA. Some people are worried in advance, but I feel like it's going to be really bad in that situation’. (F)*

#### Information considered useful to cope with knee OA

##### Evidence-based information

What participants wanted most was evidence-based information. They also wanted medical professionals to guide activities and ways of thinking based on their specialised knowledge.

*‘I can trust evidence-based information and be convinced by it. I trust that a specialist who has studied medicine properly will explain information based on scientific evidence’. (C)*

*‘I would like medical professionals to teach me how to move and how to walk properly to reduce symptoms of knee OA’. (D)*

*‘If an expert explains the pathology and mechanism theoretically, it will be easier for me to understand and to take countermeasures myself’. (G)*

##### Informal information

Participants gathered informal information about knee OA by word of mouth and media, such as magazines, TV, or the internet. Some actively collected information from these sources.

*‘I often had the opportunity to learn about the doctor's reputation and the hospital's reputation in conversations with my neighbours’. (H)*

*‘I searched for information about knee OA from TV, magazines, and books’. (E)*

#### Importance of connecting with others

##### Interacting with people in the same situation or the same generation

Participants wanted a place where they could express their painful feelings and experiences related to knee OA. They believed that such opportunities would help them psychologically. Simultaneously, they emphasised the importance of a local community of people in the same situation (facing knee OA) or people of the same generation because they did not want to bother others and thought that others, including their family, could not understand their feelings unless they were patients with knee OA.

*‘In the community, I talk about things I'm not happy with, things about my knees, and so on. I think it's really good. It's refreshing’. (A)*

*‘I think it is important to have a place where we can vent our feelings and experiences, regardless of whether our issues can be solved. It makes me feel better, encourages me, and makes me want to do my best. I don’t think the pain will be understood by others’. (E)*

*‘I think that communication between people in the same situation is effective for having a sense of calm’. (G)*

## Discussion

To our knowledge, this is the first study to explore how Japanese patients with knee OA experience and perceive knee OA during conservative treatment. In addition, it also clarified what they required to solve their problems. This study found that Japanese patients with knee OA desired evidence-based information and to connect with other people in the same situation to solve problems related to their difficulties. Participants self-analysed the causes for knee OA (Category 1) and experienced various difficulties with movement and activities in daily life (Category 2). Thereafter, they became cautious and restrained toward certain activities and developed a desire not to bother others (Category 3). Through such experiences, they found their own ways of dealing with knee OA symptoms and difficulties (Category 4). Additionally, they expressed a desire for evidence-based information (Category 5) and connections with others to eliminate both the physical and mental difficulties associated with knee OA (Category 6) (Fig. 1).

We found similarities between the perceptions of the Japanese participants in our study and those from other countries, such as the US, Canada, European countries, and some other Asian countries [20]. Causes of knee OA such as ‘Overwork/overload’ and ‘Ageing’, difficulties in daily life due to knee symptoms, psychological barriers, and the importance of connecting with others were also demonstrated in a previous systematic review [20]. Therefore, some scientific evidence from other countries could be applied to manage pain and other symptoms experienced by Japanese patients with knee OA.

Meanwhile, some characteristics were specific to Japan, such as Japanese-style movements, and related to the participants’ experiences and ideas. When self-assessing knee dysfunction, Japanese people often consider whether they can sit on their heels in a Japanese-style room. In Japan, some movements are very culture-specific, such as sitting on a tatami mat. Making such movements involves self-analysis about the causes of knee OA and the difficulties experienced in daily life. Older Japanese people often encounter such situations during group activities and ceremonial occasions. They often avoid these situations because they do not want to bother others by not being able to perform Japanese-style movements. Similar reports about culture-related activities have been documented in other countries [18, 25, 31]. In addition, a few participants thought that ‘Exposure to cold’ had caused knee OA. In Japan, people believe that exposure to cold is the cause of all illnesses; a similar explanation was also found in another study [31].

Knee OA makes patients more cautious about their activities, making them conscious of their movements and speed of movement and, in some cases, avoid movement altogether [32]. The fear avoidance model [33] states that patients’ anxiety leads to inactivity, which creates a vicious cycle that exacerbates symptoms and further increases anxiety. This was commonly reported by many participants in the current study. In addition, patients also complained of anxiety about surgery and desired to avoid it. Previous studies have also reported that patients with knee OA were anxious about joint replacement because of concerns about the effectiveness of the surgery, the risk of surgery, and the duration of recovery [14, 34–36].

From the various experiences related to knee OA as described above, participants in this study had their own beliefs and coping strategies for knee OA. One interesting finding was that participants recognised the importance of exercise and physical activity to manage symptoms of knee OA, even though they believed that past knee overuse caused the current condition of knee OA. This perspective differs from previous studies that described the belief that exercise and physical activity could increase knee pain and structural damage [31, 37, 38], despite the evidence on exercise and physical activity being recommended for patients with knee OA [5, 20, 39–41]. Other qualitative studies have also shown that people with knee OA avoid activity and exercise when they believe it will further damage their knee joint [37, 42]. In addition, the participants adopted strategies in walking and other activities of daily living to reduce knee pain and difficulty in movement. Meanwhile, participants did not have a specific criterion for judging the appropriate amount of exercise and physical activity or way of movement and used their physical sensations as criteria. Similar reports have been found in previous studies [34, 40, 43–45]; however, such self-judgement is often inaccurate [35].

Participants also recognised the importance of having a positive mindset to manage knee OA. This indicates that, if they can learn to suppress negative feelings and develop a positive attitude through appropriate guidance and information provision, it will lead to effective management of knee OA in conservative care.

Category 4 suggests that participants in this study wanted reliable, evidence-based information to understand knee OA and alleviate the various physical and mental difficulties mentioned above properly. Nonetheless, as with many previous studies [14, 19, 27, 28, 46–48], the primary information sources were informal. Previous studies in other countries demonstrated that informal rather than evidence-based information was recognised as an important resource throughout conservative care [18, 19, 31, 37]. One of the reasons why participants in this study attached importance to informal information over evidence-based information may be that they did not have sufficient opportunities to access reliable information sources and evidence-based information. Patients’ distrust of medical professionals may also be another reason [40]. A poor relationship between the medical professional and the patient at the beginning of treatment is detrimental, and mutual trust is important to foster a positive attitude toward conservative care [49]. Medical professionals should attend to patients’ complaints from the beginning to build a relationship of trust with them.

Participants in this study wanted to connect with people who were part of the same generation and had the same experiences because they believed that only such people could understand their difficulties and feeling of not wanting to bother others. Knee OA affects individuals socially because it limits their activity and participation [20, 40]. Meanwhile, peer support works in self-management interventions for people with chronic pain [50]. For example, an arthritis self-management program that included peer interaction positively affected Asian people with knee OA both physically and psychologically [51–53]. Establishing a community where people with knee OA can express their feelings and share them with peers may be effective in finding a solution for the physical and psychological barriers caused by knee OA.

This study has several limitations. First, transferability of our findings to other populations is limited. We selected sociable and expressive participants and asked for research cooperation to increase the possibility of obtaining ample data. The results obtained from extroverted people may be more positive than those obtained from introverted people. Furthermore, some patients refused to cooperate during recruitment: thus, the data obtained were related only to the patients who participated. Specifically, the results of this study are thought to reflect the experiences and perceptions of the older generation; however, perceptions and experiences may differ in younger patients, as shown in previous studies [12]. Second, the sample size was small even for a qualitative study, with only two men. The gender differences in our sample (more women) might also have affected the transferability of the results and the representativeness of the sample selection, even though this difference is consistent with the fact that more women suffer from knee OA. Further research may be needed to understand the experiences of men. Finally, the culture and living environment particular to Japanese people are reflected in the text, so caution is required when transferring the findings across cultures.

Based on the findings of this study, some clinical implications could be suggested within the limitations mentioned above. Specifically, accurately delivering patient-specific, evidence-based information is necessary. Information on activities of daily living that require knee flexion, starting movement, surgery, and the appropriate amount of activity are interpreted as patient needs. Information to reduce their difficulty of movement in Japanese-style activities and information on the effects of ‘coldness’ and how to deal with it must also be delivered to meet the needs of Japanese patients with knee OA. In addition, providing patients with the necessary and sufficient evidence-based information will reduce anxiety and increase activity. Interacting with others in the local community may also reduce the physical and psychological burden in patients with knee OA. Easy participation, forming connections, and establishing a community where reliable information can be obtained may positively affect the physical and psychological aspects of Japanese patients with knee OA patients. Though these countermeasures could be expected to have some degree of effectiveness, they need to be verified in future studies.

## Conclusions

The perceptions and beliefs of Japanese people with knee OA during conservative care have similarities and differences with those of other countries. Japanese patients with knee OA desired evidence-based information and to connect with other people in the same situation to solve problems related to their condition. Conducting careful interviews and gathering information to clarify and understand patients’ experiences related to knee OA is important. Pain and other symptoms experienced by patients with knee OA should be managed by evidence-based information integrating their perceptions and beliefs toward knee OA. Based on the results of this study, we must develop and evaluate a strategy on how to manage symptoms and disorders experienced by patients with knee OA in the future.

## Supplementary Information

**Additional file 1.** Illustrative quotes by category. Illustrative quotes from interview data.

## Data Availability

The datasets used and/or analysed during the current study are available from the corresponding author on reasonable request.

## Abbreviations

OA: Osteoarthritis
TKA: Total Knee Arthroplasty
