# Supplementary material for: Perceptions, beliefs, and needs of Japanese people with knee osteoarthritis during conservative care: a qualitative study
Source: BMC Musculoskelet Disord. 2021 Sep 3;22:754. doi: 10.1186/s12891-021-04641-7 (PMC8417949; doi:10.1186/s12891-021-04641-7)
Supplement: Supplementary file 1 — Additional file 1. Illustrative quotes by category. Illustrative quotes from interview data. [file 12891_2021_4641_MOESM1_ESM.docx]

Additional file: Illustrative quotes by category

The categories are presented first followed by subcategories. The subcategories were described with illustrative quotes which were presented throughout the text using pseudonyms that matches table 2.

***1. Self-analysis on the cause of knee OA***

1. overwork/overload

“When I was very busy with work, I had other people help me, but I was the one who carried all the things. I think I was forced to move too much.” (B)

“I had been helping my husband, who had been self-employed since he was about 40 years old, but if I hadn't helped my husband and carried a lot of heavy things, I think my knees wouldn't have been so bad. If I were a full-time housewife, I wouldn't have suffered so much pain.” (E)

“I had a sudden onset, but I was busy about housework before that, so I thought it was because I moved too much.” (G)

“I thought I had to walk for exercise, but I think I walked too much.” (B)

“I think my knees got worse not because of my age, but because I walked too much. I think it's overdone.” (C)

“Considering from the results of gait analysis by a physiotherapist, I wondered if there was a relationship between the characteristics of walking and osteoarthritis.” (F)

“I have a feeling that the usual way of walking accumulates a burden on the knees and manifests itself in symptoms.” (G)

“I have had slight deformity in my ankle since I was a child, and I think that is also related to the condition of my knee.” (H)

2. Aging

“I think the one of the reasons why my knees got worse is aging.” (D)

“I think it may be unavoidable that osteoarthritis develops because of aging.” (F)

“It was said that knee OA was due to age, and I wondered if it couldn't be helped. However, because the doctor said that, I thought it couldn't be helped anymore.” (B)

“I have no idea that my knees will not improve because I am old. I don't think it's because I'm old that my knee symptoms don't improve.” (I)

3. Exposure to cold

“I think that coldness is the cause of pain in addition to age.” (C)

“I think the reason why my knees got worse is that I was cold in the morning. I don't think it's because I moved my knees too much, but in my case it was because my knees were cold.” (D)

“In the car I was riding at work, the wind from the air conditioner hit my knees directly. So in my case, I think one of the reasons my knees got worse was that my knees were absolutely cold, though I don’t know about others.” (E)

“My knees ache on cold days” (E)

***2. Difficulty in daily life due to knee symptoms***

1. Movement with bending knees

“My knees hurt when going up and down stairs and getting in and out of the car.” (B)

“It wouldn't hurt so much if I was standing, but I couldn't crouch down and work.” (B)

“It is difficult to go down the stairs.” (F)

“Though I can't do it on level ground like I used to, I can walk slowly. However, it is very difficult to walk on the slope. It is especially difficult to walk downhill than uphill.” (I)

“It's hard to climb the stairs, but it's more difficult to get down. I can't get off without a handrail.” (I)

“I can't use Japanese-style toilets.” (A)

“I cannot sit down on my heels in Japanese room.” (G)

2. Start of movement

“I felt pain when I got up from the chair.” (H)

***3. Psychological barrier***

1. Prudence about movement and activities

“I'm more cautious than before when moving. because I think it would be a problem if I don't have enough strength or fall.” (F)

“I'm trying not to run or hurry as much as possible.” (G)

“I think it is important to be careful about how you move and walk.” (G)

“I had a pain in my knee, so I couldn't move too much. I tried not to move much in my daily life. (H)

“I used to try everything myself without relying on people, but I gave up it.” (I)

2. Not wanting bother others

“I think people don't like hearing someone complaining.” (G)

“Rather than not wanting to take a break from work or teaching Tai Chi, I didn't want to bother with taking a break.” (I)

3. Desire to avoid surgery

“I was afraid to have surgery, so I took painkillers, gave injections, and used all means to prolong the surgery.” (B, E)

***4. How to deal with knee pain and difficulty in moving***

1. Controlling activities based on subjective sensations

“After I hurt my knee, I often turned my attention to my body (sense).” (I)

“I can move as far as I can. If I can't move, stop.” (I)

“If we feel that our knees are in a different condition than usual, we should go to the hospital immediately.” (F)

“If you feel pain, you shouldn't put up with it or divert your consciousness. It's a problem that it hurts too much, but I think it's a warning that something is wrong.” (G)

“If you start to feel pain in your knees, stop walking.” (C)

“I felt pain in my knees on a rainy day. Even if I walked, when it became cloudy or when it was raining, I couldn't walk the distance I always walked.” (B)

“It's difficult to adjust the amount of exercise, because I didn't have any knee pain at all, but it gradually becomes painful.” (B)

2. Continuing exercise and physical activity

“I devised myself to strengthen muscles to improve the condition of my knees, and I pay attention to my diet to strengthen my bones.” (I)

“I want to tell myself in the past that I should exercise firmly. I think exercise is important.” (H)

“I think it's better to exercise than not to do. I think it is important to keep your physical activity to some extent. Don’t be lazy.” (F)

“When I go out and come back, my knees are in good condition. Based on my experience, I think it's good for your knees to move.” (G)

“I think it would be better to move my joints, so I continue to do light exercise.” (G)

“I consciously walk every day and try to use the stairs as much as possible instead of the elevator or escalator.” (B)

“I think we should walk moderately. My knees hurt more when I wasn't moving. The condition of my knees was better when I walked to some extent.” (B)

“I ride a bicycle for exercise, but after about 15 minutes, my knees bend well. Your knees will improve.” (B)

“I go to the field work while inspiring myself.” (I)

“When I had a pain in my knee, I felt that if I didn't move, the pain would have become stronger, so I try to go out as much as possible. I try to go to places with slopes rather than flat ground.” (I)

“We volunteer to clean the shrine every morning.” (C and D)

“I try to be active every day without worrying about pain.” (C)

3. Ingenuity to reduce knee pain and difficulty in movement

“When doing work such as lifting and lowering heavy luggage, I divide the movements one by one so that my knees are not strained.” (C)

“I am careful not to sit down on my heels.” (G)

“When I walk on the stairs, I hurt my knees when I walk alternately, so I try to walk step by step.” (C)

“I stopped wearing high heels.” (F)

“I know for myself that it's okay to move in this way.” (C)

“It is absolutely not good to bend your knees and work in the fields. Work while sitting with a cushion or something like a chair.” (D)

“I try to walk the stairs carefully. At stations and trains, I always try to stand on the railing.” (G)

4. Way of thinking

“I think it has something to do with how the person has lived and in what environment he/she has lived” (E)

“I think it's good to be motivated and work on various things. I think it's better to look for what you can do with a positive feeling. I think people will change depending on how they think.” (E)

“It's very painful to start worrying about knee pain, so I try not to think about it.” (C)

“Looking at the other people who have improved, I also hope to be like that. If I want to be that way, I think my condition will improve.” (I)

***5. Information that is considered useful to cope with knee OA***

1. Evidence-based information

“I think it would be better to know how to exercise, how long the knee condition generally goes, and the healing process from medical professionals.” (A)

“There were many questions that I would like experts to tell me when I visited the clinic.” (B)

“I think we should ask an expert to get a solution.” (G)

“It would be great if there was a place where participants could exchange information and communicate while exercising together with the guidance of a medical specialist such as a physiotherapist.” (H)

“I wanted doctors and physiotherapists to tell me what to do depending on the condition, and if there is a way to slow down the progression of knee OA.” (H)

“I trust the advice from my daughter who works as a nurse.” (I)

2. Informal information

“I am very interested in the information that my friends and acquaintances have.” (I)

“I tried everything that my acquaintance taught me at least once.” (B)

“I don't like the trendy gymnastics featured on TV, and I'm not much influenced by it. “(C)

“If TV program happen to be dealing with health information while watching TV, I will see it, but at most that's about it.” (F)

***6. Importance of connecting with others***

1. Interacting with people in the same situation or in the same generation

“I think that social connection in the community play a role in supporting our health including the issue about knee OA.” (F)

“If an expert explains the pathology and mechanism theoretically, it will be easier for me to understand and to take countermeasures myself.” (G)

“Some people, even those who have never experienced knee pain, irresponsibly give their opinions.” (B)

“I think it's important to get dressed, go out and talk to other people.” (I)
